# Supplementary material for: Translation factor eIF5a is essential for IFNγ production and cell cycle regulation in primary CD8+ T lymphocytes
Source: Nat Commun. 2022 Dec 17;13:7796. doi: 10.1038/s41467-022-35252-y (PMC9759561; doi:10.1038/s41467-022-35252-y)
Supplement: Supplementary file 1 — Supplementary Information [file 41467_2022_35252_MOESM1_ESM.pdf]

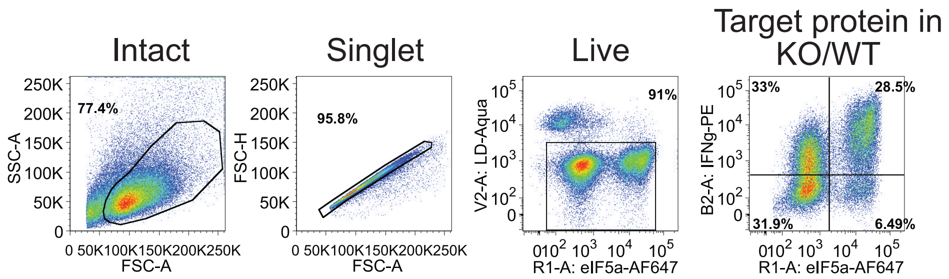

Supplementary Figure 1. Representative gating strategies for all flow cytometry experiments.

Cells were first gated on forward and side scatter (FSC/SSC) to include only intact cells. For doublet exclusion, singlets were defined as the main population in FSC-Area/FSC-Height. Live cells were defined as LIVE/DEAD™ Aqua-negative. Where possible, KO/WT cells were separated using antibody against eIF5a or hypusine, and intensity of target protein(s) was monitored individually in the two populations.

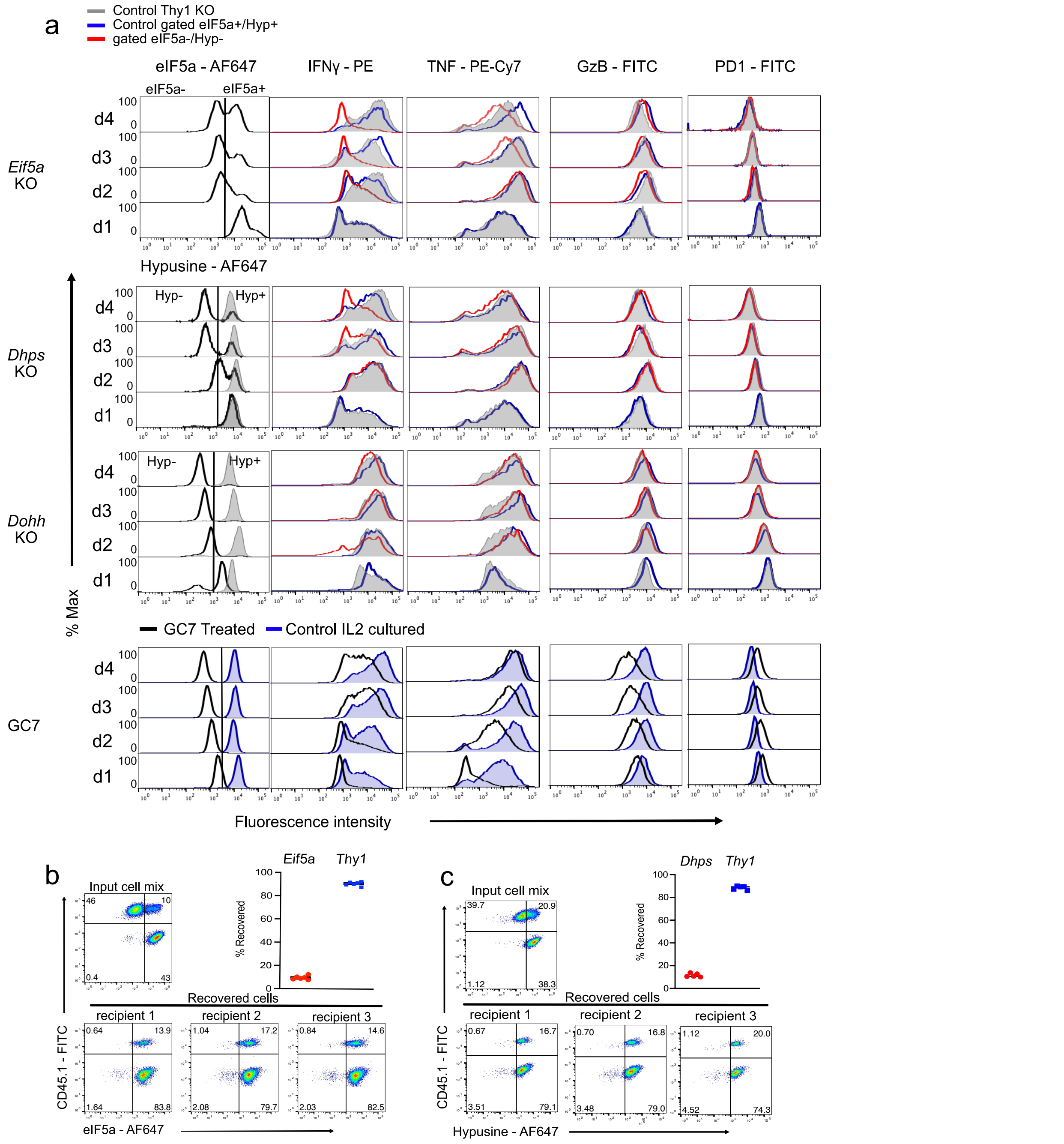

Supplementary Figure 2. Time course of protein reduction following CRISPR knockout and in vivo response of KO cells to LmOVA infection.
a Expression of eIF5a or hypusine was followed, together with the abundance of IFNγ, TNF, Granzyme B, and PD-1, over 4d following CRISPR KO. Electronically gated eIF5a/hypusine+ (blue line) and eIF5a/hypusine- (red line) populations in the same sample were overlaid, together with the *Thy1* CRISPR control (grey line shaded). GC7-treated (black line) samples were overlaid with their mock-treated (blue line shaded) controls. One representative of 3 independent experiments is shown. **b** *Eif5a* or **c** *Dhps* CRISPR cells (CD45.1) were mixed with equal number of *Thy1* CRISPR cells (CD45.2), and injected into CD45.1/CD45.2 female C57BL/6 WT mice 8-10 weeks of age together with *Listeria monocytogenes* expressing chicken ovalbumin (LmOVA). Analysis gated on the donor cells using CD45 allele-specific Abs, shows the input cell mix and the recovered cells at d6 from 3 independent recipients. The recovered proportions from all mice in the experiment are plotted on the right. *eIF5a* KO n=6 biological replicates each from 2 independent experiments and *Dhps* KO n=5 biological replicates from a single experiment .

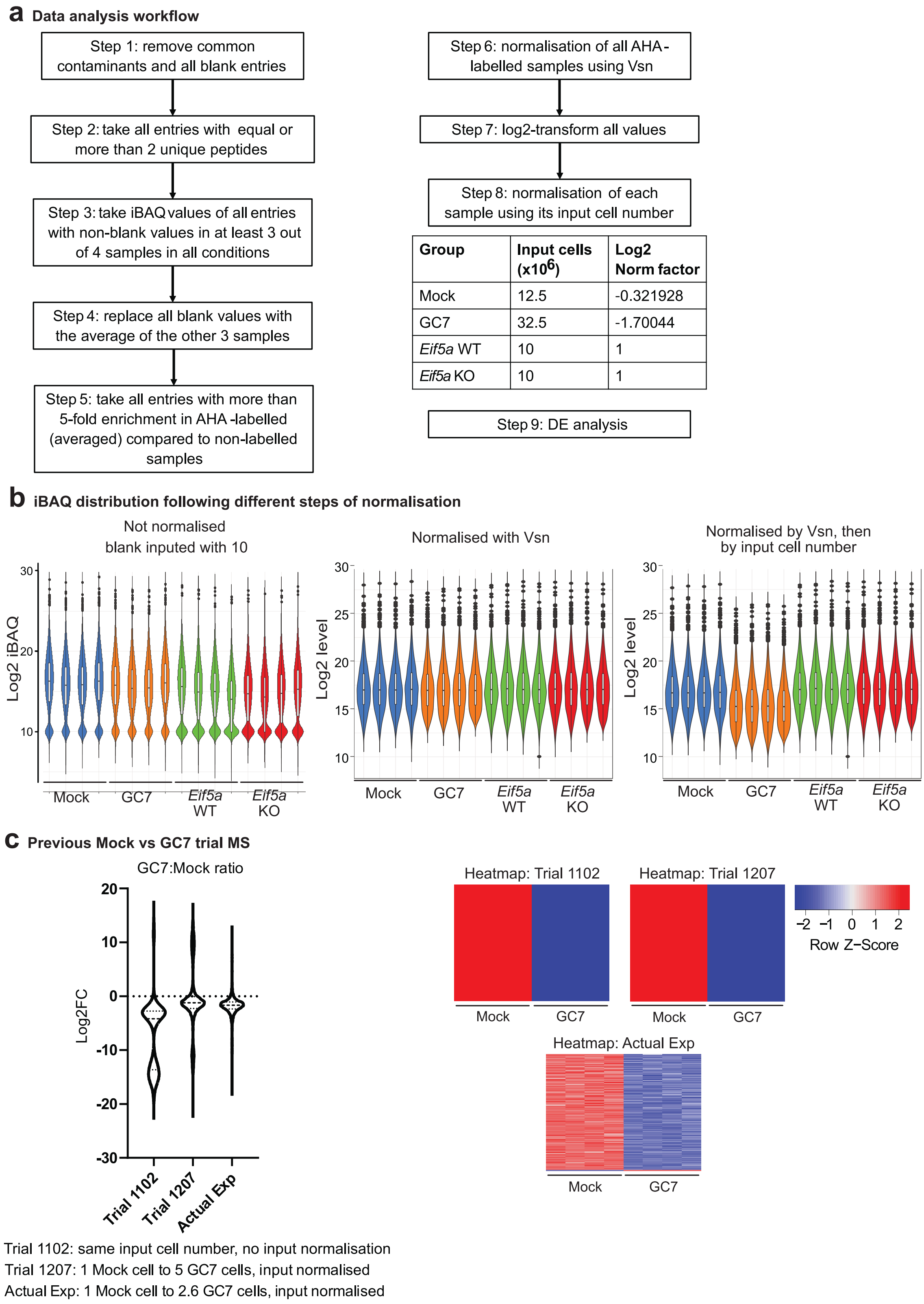

Group	Input cells (x10^6)	Log2 Norm factor
Mock	12.5	-0.321928
GC7	32.5	-1.70044
Eif5a WT	10	1
Eif5a KO	10	1

Supplementary Figure 3. Normalisation of nascent proteome dataset, and comparison with previous trial experiments.
a Workflow illustrating how expression cut off was set and how iBAQ values were processed and normalised. **b** Violin plots showing log2 iBAQ of 3246 proteins detected in 3 of 4 samples without any normalisation, after Vsn normalisation, and after further normalisation by input cell number. Center line, median; box limits, upper and lower quartiles; whiskers, 1.5x interquartile range; points, outliers. Colours: blue, Mock; orange, GC7; green, *Eif5a* WT; red, *Eif5a* KO. **c** Violin plot showing log2 GC7:Mock ratios in two previous trial experiments (1102: same number of input cells between Mock- and GC7-treated samples, n=1; 1207: 1 Mock cell to 5 GC7 cells, with input normalisation, n=1) and in the actual experiment reported here (with input normalisation, n=4). Heat maps[1] showing log2 normalised iBAQ of 2500 most different proteins in the three experiments are shown below.

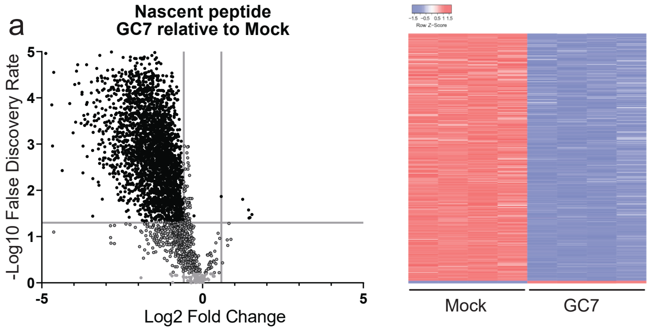

Top 10 enriched GO terms in Molecular Function	
**Down-regulated: 2617 proteins**	**Up-regulated: 5 proteins**
translation factor activity, RNA binding	Ndufa7, NADH dehydrogenase [ubiquinone] 1 alpha subcomplex subunit 7
disordered domain specific binding	Mrps6, 28S ribosomal protein S6, mitochondrial
phospholipid binding	Phb1, Prohibitin 1
endopeptidase activity	Foxo3, Forkhead box protein O3
enzyme activator activity	Rpl38, 60S ribosomal protein L38
ligase activity	
amide binding	
structural constituent of cytoskeleton	
oxidoreductase activity, acting on the CH-CH group of donors	
oxidoreductase activity, acting on CH-OH group of donors	

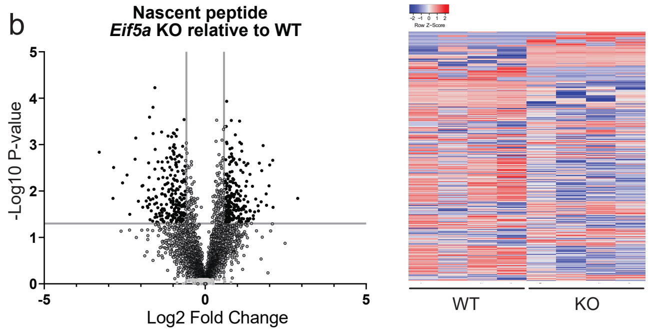

Top 10 enriched GO terms in Molecular Function	
**Down-regulated: 234 proteins**	**Up-regulated: 216 proteins**
actinin binding	structural constituent of ribosome
coenzyme binding	rRNA binding
ligase activity	oxidoreductase activity, acting on NAD(P)H
NF-kappaB binding	oxidoreductase activity, acting on the aldehyde or oxo group of donors
damaged DNA binding	phosphotransferase activity, phosphate group as acceptor
protein kinase C binding	nucleobase-containing compound kinase activity
cell adhesion molecule binding	dynein light intermediate chain binding
hormone receptor binding	chaperone binding
ubiquitin-like protein ligase binding	carbohydrate binding
oxidoreductase activity, acting on paired donors, with incorporation or reduction of molecular oxygen	GTPase activity

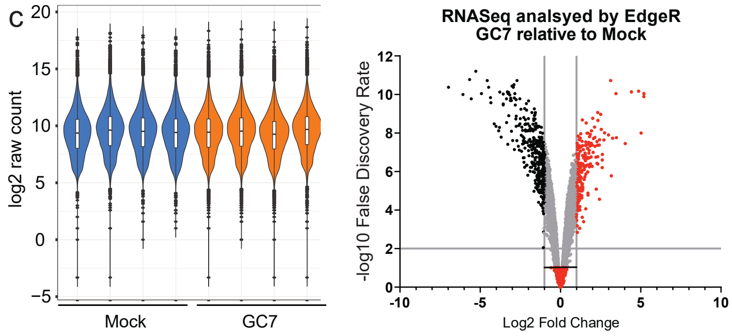

Supplementary Figure 4. Differential expression analyses of nascent proteins

a GC7-treated or **b** *Eif5a* KO volcano plots of the nascent peptide dataset using the significance criterion FDR or P-value<0.05, Absolute FC>2. FDRs were calculated using unpaired two-sided T test with Benjamini-Hochberg correction, P values were calculated using paired two-sided T test. Heat maps were constructed using the top 2500 significantly changed proteins [1], relative protein abundance is graded from low (blue), medium (white) to high (red) to allow comparisons between samples. Top 10 enriched GO terms in molecular function were generated using WebGestalt [2]. **c** Distribution of log2 raw counts for 11183 transcripts from individual replicates of RNAseq data of 4 mock and 4 GC7 treated cells at Day 2. Center line, median; box limits, upper and lower quartiles; whiskers, 1.5x interquartile range; points, outliers. Colours: blue, Mock; orange, GC7. Volcano plot of differentially expressed genes is shown on the right, with reduced genes (black dots) defined as FDR<0.01, FC<-2; and "not-reduced" genes (red dots) defined using the criteria FDR>0.1 or FDR<0.01 while FC>2. FDRs were calculated with edgeR.

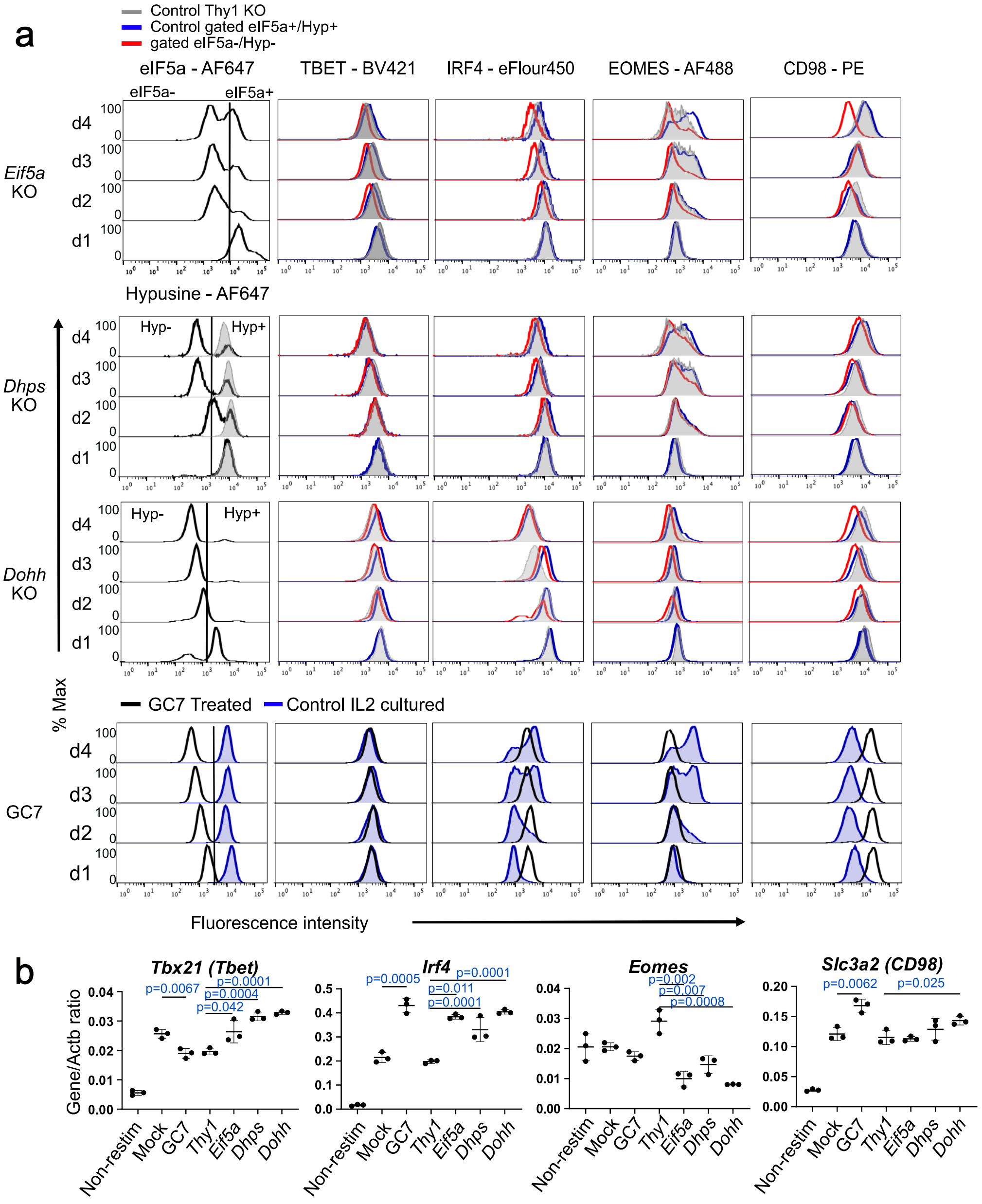

Supplementary Figure 5. Time course of expression of key transcription factors and cell surface receptor CD98 following GC7 treatment or CRIPSR transfection.
a FACS overlay histograms of electronically gated eIF5a/Hyp+ (blue line) and eIF5a/hyp- (red line) populations together with control Thy 1 KO (grey line shaded) over 4d following CRISPR targetting. GC7-treated samples (black line) were overlaid with their mock-treated controls (blue line shaded). One representative of 3 independent experiments is shown. In each of the 4 days cells were fixed and permeabilised as described in Methods (CD98 were stained before permeabilisation), and stained with target-specific antibodies together with antibody against eIF5a or hypusine, where applicable. The leftmost knockout histograms are identical to Supplementary Figure 2 as the same samples were used, but with different Ab staining panels for the proteins of interest. **b** RT-qPCR quantitation of mRNA for the same genes, normalised by the mRNA level of Actb gene, in fresh bulk transfected cells. For Mock, GC7, *Dhps*, *Dohh*, and *Thy1* KO Day 4 cells were used, and for *Eif5a* KO Day 3 cells were used (n=3 biological replicates. P values were calculated using unpaired two-sided T test, GC7 compared to Mock and CRISPR cells compared to Thy1). Centre line and error bars represent mean value ± SD between biological replicates.

References:

1. Babicki, S., *et al.* Heatmapper: web-enabled heat mapping for all. *Nucleic acids research* **44**, W147-153 (2016).
2. Liao, Y., Smyth, G.K. & Shi, W. featureCounts: an efficient general purpose program for assigning sequence reads to genomic features. *Bioinformatics* **30**, 923-930 (2013)

Supplementary Table 1 | Oligonucleotides (custom synthesised by Integrated DNA Technologies)

Gene	Forward primer	Reverse primer
Actb	ATGGAGGGGAATACAGCCC	TTCTTTGCAGCTCCTTCGTT
Ifng	GAGCTCATTGAATGCTTGGC	GCGTCATTGAATCACACCTG
Tnf	CTGAACTTCGGGGTGATCGG	GGCTTGTCACTCGAATTTTGAGA
Irf4	TCCGACAGTGGTTGATCGAC	CCTCACGATTGTAGTCCTGCTT
Tbx21 (Tbet)	AACACACACGTCTTTACTTTCCA	CGTATCAACAGATGCGTACATGG
Eomes	GACCTCCAGGGACAATCTGA	GTGACGGCCTACCAAAACAC
Cdk1	AGAAGGTACTTACGGTGTGGT	GAGAGATTTCCCGAATTGCAGT
Slc3a2 (CD98)	TGATGAATGCACCCTTGTACTTG	GCTCCCCAGTGAAAGTGGA
