# Supplementary material for: Translation factor eIF5a is essential for IFNγ production and cell cycle regulation in primary CD8+ T lymphocytes
Source: Nat Commun. 2022 Dec 17;13:7796. doi: 10.1038/s41467-022-35252-y (PMC9759561; doi:10.1038/s41467-022-35252-y)
Supplement: Supplementary file 3 — Description of Additional Supplementary Files [file 41467_2022_35252_MOESM3_ESM.pdf]

Description of Additional Supplementary Files

File Name: Supplementary Data 1
Description: **Normalised iBAQ values of nascent proteomes, differential expression analysis, and selection of proteins for presentation.** iBAQ values of expressed proteins from LC-MS for GC7-treated, Mock-treated, eIF5a-KO and eIF5a-WT cells were normalised using Vsn algorithm, and then by the input cell numbers. Statistical tests were performed using log2-transformed normalised values. Unpaired T test with Benjamini-Hochberg correction was used to determine significantly down-regulated proteins between GC7- and Mock-treated samples. Paired t-test was used for eIF5a KO versus WT samples.

File Name: Supplementary Data 2
Description: **Differential expression analysis of the RNASeq dataset between GC7- and Mock-treated cells, with top ten enriched KEGG pathways in significantly up- and down-regulated genes.** The dataset was trimmed of adapter sequences and low quality reads were removed. Reads were aligned to the mouse genome (GRCm38.96) using STAR and mapped reads were counted using featureCounts. Differential expression analysis was performed with edgeR. Pathway enrichment analysis was conducted with WebGestalt.

File Name: Supplementary Data 3
Description: **Determination of genes translationally regulated by eIF5a using Venn analyses.** Genes translationally down-regulated upon GC7-treatment were defined as those not down-regulated in mRNA abundance but decreased in nascent peptide abundance following treatment. This list of genes was overlaid with nascent proteins down-regulated in eIF5a-KO cells and the common genes between them were defined as genes translationally regulated by eIF5a.
