# Supplementary material for: Translation factor eIF5a is essential for IFNγ production and cell cycle regulation in primary CD8+ T lymphocytes
Source: Nat Commun. 2022 Dec 17;13:7796. doi: 10.1038/s41467-022-35252-y (PMC9759561; doi:10.1038/s41467-022-35252-y)
Supplement: Supplementary file 7 — Reporting Summary [file 41467_2022_35252_MOESM7_ESM.pdf]

nature portfolio

Corresponding author(s): Rose Zamoyska

Last updated by author(s): 13/11/2022

# Reporting Summary

Nature Portfolio wishes to improve the reproducibility of the work that we publish. This form provides structure for consistency and transparency in reporting. For further information on Nature Portfolio policies, see our Editorial Policies and the Editorial Policy Checklist.

## Statistics

For all statistical analyses, confirm that the following items are present in the figure legend, table legend, main text, or Methods section.

| n/a | Confirmed | |
|---|---|---|
| ☐ | ☒ | The exact sample size (*n*) for each experimental group/condition, given as a discrete number and unit of measurement |
| ☐ | ☒ | A statement on whether measurements were taken from distinct samples or whether the same sample was measured repeatedly |
| ☐ | ☒ | The statistical test(s) used AND whether they are one- or two-sided<br>*Only common tests should be described solely by name; describe more complex techniques in the Methods section.* |
| ☒ | ☐ | A description of all covariates tested |
| ☐ | ☒ | A description of any assumptions or corrections, such as tests of normality and adjustment for multiple comparisons |
| ☐ | ☒ | A full description of the statistical parameters including central tendency (e.g. means) or other basic estimates (e.g. regression coefficient) AND variation (e.g. standard deviation) or associated estimates of uncertainty (e.g. confidence intervals) |
| ☐ | ☒ | For null hypothesis testing, the test statistic (e.g. *F*, *t*, *r*) with confidence intervals, effect sizes, degrees of freedom and *P* value noted<br>*Give P values as exact values whenever suitable.* |
| ☒ | ☐ | For Bayesian analysis, information on the choice of priors and Markov chain Monte Carlo settings |
| ☒ | ☐ | For hierarchical and complex designs, identification of the appropriate level for tests and full reporting of outcomes |
| ☒ | ☐ | Estimates of effect sizes (e.g. Cohen's *d*, Pearson's *r*), indicating how they were calculated |

*Our web collection on statistics for biologists contains articles on many of the points above.*

## Software and code

Policy information about availability of computer code

| | |
|---|---|
| Data collection | Cutadapt v2.3, fastqc v0.11.8, STAR v2.7.1a and samtools v1.9 (updated 17th June 2019) used for pre-treatment of RNASeq files; rtracklayer v1.42.2, Rsubread v1.6.4 in R (updated 19th June 2019) used for collecting gene expression data in RNASeq; MaxQuant version 1.6.2.6 used to collect expression data in nascent proteomic dataset. |
| Data analysis | EdgeR v3.26.4 in R (updated 20th June 2019) used for normalisation and differential expression analysis of RNASeq. Vsn algorithm used for normalisation, and T tests and BH correction for differential expression of proteomic dataset. T tests used for flow cytometry and qRT-PCR data done in Excel 2016 and Prism 9. Flowjo version 9 was used to analyse flow cytometry data. |

For manuscripts utilizing custom algorithms or software that are central to the research but not yet described in published literature, software must be made available to editors and reviewers. We strongly encourage code deposition in a community repository (e.g. GitHub). See the Nature Portfolio guidelines for submitting code & software for further information.

## Data

Policy information about availability of data

All manuscripts must include a data availability statement. This statement should provide the following information, where applicable:

- Accession codes, unique identifiers, or web links for publicly available datasets
- A description of any restrictions on data availability
- For clinical datasets or third party data, please ensure that the statement adheres to our policy

Source data and codes are available on Open Science Framework at DOI 10.17605/OSF.IO/J94BY

Original and processed RNASeq dataset have been deposited in the Gene Expression Omnibus (GEO) database, https://www.ncbi.nlm.nih.gov/geo (accession no.

GSE168731).

Original files for the nascent proteomic dataset are available at EBI PRIDE database, https://www.ebi.ac.uk/pride/ (accession no. PXD021063).

# Field-specific reporting

Please select the one below that is the best fit for your research. If you are not sure, read the appropriate sections before making your selection.

☒ Life sciences ☐ Behavioural & social sciences ☐ Ecological, evolutionary & environmental sciences

For a reference copy of the document with all sections, see nature.com/documents/nr-reporting-summary-flat.pdf

# Life sciences study design

All studies must disclose on these points even when the disclosure is negative.

| | |
|---|---|
| Sample size | Sample size per group was calculated as 4 for reliably detecting eIF5a inhibition. This was calculated using ClinCalc: https://clincalc.com/stats/samplesize.aspx.<br>With preliminary data indicating WT eIF5a mean of 0.864070016 and inhibited mean of 0.417720874,<br>Alpha = 0.05, Beta = 0.2, Power = 0.8. |
| Data exclusions | No data was excluded. |
| Replication | n number for each experiment are stated in figure legend. All replicates were successfully conducted. |
| Randomization | Sex of animals used for tissue donation was randomised as difference between sexes was not anticipated. |
| Blinding | Blinding was not relevant as the in vitro knockout samples and subsequent analysis were conducted by the same experimentalist. In the animal experiments cells were co-transferred into a single recipient so all biological replicates were identical. |

# Reporting for specific materials, systems and methods

We require information from authors about some types of materials, experimental systems and methods used in many studies. Here, indicate whether each material, system or method listed is relevant to your study. If you are not sure if a list item applies to your research, read the appropriate section before selecting a response.

## Materials & experimental systems

| n/a | Involved in the study | |
|---|---|---|
| ☐ | ☒ | Antibodies |
| ☒ | ☐ | Eukaryotic cell lines |
| ☒ | ☐ | Palaeontology and archaeology |
| ☐ | ☒ | Animals and other organisms |
| ☒ | ☐ | Human research participants |
| ☒ | ☐ | Clinical data |
| ☒ | ☐ | Dual use research of concern |

## Methods

| n/a | Involved in the study | |
|---|---|---|
| ☒ | ☐ | ChIP-seq |
| ☐ | ☒ | Flow cytometry |
| ☒ | ☐ | MRI-based neuroimaging |

## Antibodies

| | |
|---|---|
| Antibodies used | eIF5a clone EP527Y Abcam ab32407; RRID:AB_732132; used at 1:400 for Flow cytometry and 1:1000 for Western blot.<br>Hypusine clone Hpu24 Creative Biolabs PABL-202; used at 1:200 for Flow cytometry and 1:1000 for Western blot.<br>IFNg clone XMG1.2 (PE conjugated) BioLegend 505808; RRID:AB_315402; used at 1:400<br>TNFa clone MP6-XT22 (PE-Cy7 conjugated) eBioscience 25-7321-82; RRID:AB_11042728; used at 1:400<br>IRF4 clone 3E4 (eFlour 450 conjugated) eBioscience 48-9858-82; RRID:AB_2574135; used at 1:200<br>TBET clone 4B10 (Brilliant Violet 421 conjugated) BioLegend 644832; RRID:AB_2686976; used at 1:200<br>EOMES clone Dan11mag (Alexa Fluor® 488 conjugated) Thermo Scientific 53-4875-82; RRID: AB_10854265; used at 1:200<br>Granzyme B clone GB11 (FITC conjugated) BioLegend 515403; RRID: AB_2114575; used at 1:200<br>CDK1 clone A17 Abcam ab18; RRID:AB_2074906; used at 1:200<br>CD25 clone PC61 (PE-Cy7 conjugated) BioLegend 102016; RRID:AB_312865; used at 1:200<br>CD98 clone RL388 (PE conjugated) eBioscience 12-0981-83; RRID: AB_465792; used at 1:200<br>CD279 (PD-1) clone RMP1-30 (FITC conjugated) eBioscience 11-9981-82; RRID: AB_465467; used at 1:200<br>CDC45 clone EPR5759 Abcam ab126762; RRID:AB_11140216; used at 1:200<br>Puromycin clone 12D10 (Alexa Fluor® 488 conjugated) Millipore MABE343-AF488; RRID:AB_2736875; used at 1:500<br>Ki-67 clone B56 (PE conjugated) BD Biosciences 556027; RRID:AB_2266296; used at 1:10<br>Goat anti-rabbit IgG (Alexa Fluor 647 conjugated) Thermo Fisher Scientific A27040; RRID:AB_2536101; used at 1:500<br>Rat anti-mouse IgG2a clone RMG2a-62 (PE conjugated) BioLegend 407108; RRID: AB_10549456; used at 1:200<br>ZAP70 clone 29 BD Biosciences 610239; RRID:AB_397634; used at 1:1000 |


March 2021

DHPS Abcam ab224134; used at 1:1000
Goat anti-Rabbit IgG (IRDye® 680RD conjugated) LI-COR Biosciences 926-68071; RRID:AB_10956166; used at 1:5000
Goat anti-Mouse IgG (IRDye® 800CW conjugated) LI-COR Biosciences 926-32210; RRID:AB_621842; used at 1:5000

Validation

1. eIF5a clone EP527Y Abcam ab32407. Validated by manufacturer and 3 publications for Flow cytometry, Wetsen blot and Immunohistochemistry. Validated by our Eif5a CRISPR mouse T cells.
2. Hypusine clone Hpu24 Creative Biolabs PABL-202. Validated by manufacturer for ELISA and by a publication for Western blot. Validated for Flow cytometry by our Dhps and Dohh CRISPR mouse T cells.
3. IFNg clone XMG1.2 (PE conjugated) BioLegend 505808. Validated by manufacturer and by 98 publications for Flow cytometry in mouse cells.
4. TNFa clone MP6-XT22 (PE-Cy7 conjugated) eBioscience 25-7321-82. Validated by manufacturer and by 30 publications for Flow cytometry in mouse and human cells.
5. IRF4 clone 3E4 (eFlour 450 conjugated) eBioscience 48-9858-82. Validated by manufacturer and 13 publications for Flow cytometry in mouse and human cells.
6. TBET clone 4B10 (Brilliant Violet 421 conjugated) BioLegend 644832. Validated by manufacturer and 28 publications for Flow cytometry in mouse and human cells.
7. EOMES clone Dan11mag (Alexa Fluor® 488 conjugated) Thermo Scientific 53-4875-82. Validated by manufacturer and 35 publications for Flow cytometry in mouse and human cells.
8. Granzyme B clone GB11 (FITC conjugated) BioLegend 515403. Validated by manufacturer and 58 publications for Flow cytometry in mouse and human cells.
9. CDK1 clone A17 Abcam ab18. Validated by manufacturer and 114 publications for Flow cytometry, Wetsen blot and Immunohistochemistry in human and mouse cells.
10. CD25 clone PC61 (PE-Cy7 conjugated) BioLegend 102016. Validated by manufacturer and by 38 publications for Flow cytometry in mouse cells.
11. CD98 clone RL388 (PE conjugated) eBioscience 12-0981-83. Validated by manufacturer and by 8 publications for Flow cytometry in mouse cells.
12. CD279 (PD-1) clone RMP1-30 (FITC conjugated) eBioscience 11-9981-82. Validated by manufacturer and by 9 publications for Flow cytometry in mouse cells.
13. CDC45 clone EPR5759 Abcam ab126762. Validated by manufacturer and by 3 publications for Western blot, Immunohistochemistry and Immunocytochemistry in human cells.
14. Puromycin clone 12D10 (Alexa Fluor® 488 conjugated) Millipore MABE343-AF488. Validated by manufacturer and by 14 publications for Flow cytometry, Western blot and Immunofluorescence in puromycin-labelled human and mouse cells.
15. Ki-67 clone B56 (PE conjugated) BD Biosciences 556027. Validated by manufacturer and 14 publications for Flow cytometry in mouse and human cells.
16. ZAP70 clone 29 BD Biosciences 610239. Validated by manufacturer and by 5 publications for Western blot, Immunofluorescence and Immunohistochemistry in Human, Mouse, Rat and Chicken cells.
17. DHPS Abcam ab224134. Validated by manufacturer for Western blot, Immunohistochemistry and Immunocytochemistry in human cells.

# Animals and other organisms

Policy information about studies involving animals; ARRIVE guidelines recommended for reporting animal research

Laboratory animals

Mice expressing the OT-1 TCR transgene (C57BL/6-Tg(TcraTcrb)1100Mjb/J) backcrossed to the Rag-1KO (B6.129S7-Rag1tm1Mom/J) background and containing congenic alleles for CD45.2 or CD45.1 were used as LN donors for CD8 T cells in all experiments. Mice were between 5 and 12 weeks of age and sexes were randomised for tissue donors. WT (CD45.1xCD45.2) C57BL/6J (Charles River) mice were used as recipients for in vivo experiments. All mice were bred and housed in individually ventilated cages under specific pathogen-free conditions at the University of Edinburgh Bioresearch and Veterinary Services (BVS) facilities.

Wild animals

Not applicable

Field-collected samples

Not applicable

Ethics oversight

This study was approved by the Ethical Review Body at the School of Biological Sciences, University of Edinburgh. All animal experiments were approved by the University of Edinburgh Bioresearch and Veterinary Services Ethical Review body and the United Kingdom Home office under project licence P38881828 to RZ.

Note that full information on the approval of the study protocol must also be provided in the manuscript.

# Flow Cytometry

## Plots

Confirm that:

☒ The axis labels state the marker and fluorochrome used (e.g. CD4-FITC).

☒ The axis scales are clearly visible. Include numbers along axes only for bottom left plot of group (a 'group' is an analysis of identical markers).

☒ All plots are contour plots with outliers or pseudocolor plots.

☒ A numerical value for number of cells or percentage (with statistics) is provided.

## Methodology

| | |
|---|---|
| Sample preparation | Cells were labelled with LIVE/DEAD™ Aqua Dead Cell Stain Kit (Invitrogen) and then fixed in Intracellular Staining Fixation Buffer (BioLegend) and permeabilised in Intracellular Staining Permeabilization Wash Buffer (BioLegend). Cells were incubated with primary antibodies for 16 hours at 4°C, washed, followed by staining with Goat anti-rabbit Alexa Fluor 647 secondary antibody (Thermo Fisher Scientific) for 1 hour at RT. |
| Instrument | Analysis was done on MACSQuant flow cytometer (Miltenyi Biotec); cell sorting was done on FACS Aria (BD). |
| Software | Flowjo version 9 was used to analyse flow data. |
| Cell population abundance | CD8 T cells were more than 99% of dissected lymphocytes. For CRISPR knockout, KO cells ranged from 50-80% in culture. |
| Gating strategy | Intact cells based on FSC-SSC, Singlets based on FSC-H and FSC-A, Life-Dead Aqua -ve. |

☒ Tick this box to confirm that a figure exemplifying the gating strategy is provided in the Supplementary Information.
