# Supplementary figures and images for: Translation factor eIF5a is essential for IFNγ production and cell cycle regulation in primary CD8+ T lymphocytes
Source: Nat Commun. 2022 Dec 17;13:7796. doi: 10.1038/s41467-022-35252-y (PMC9759561; doi:10.1038/s41467-022-35252-y)

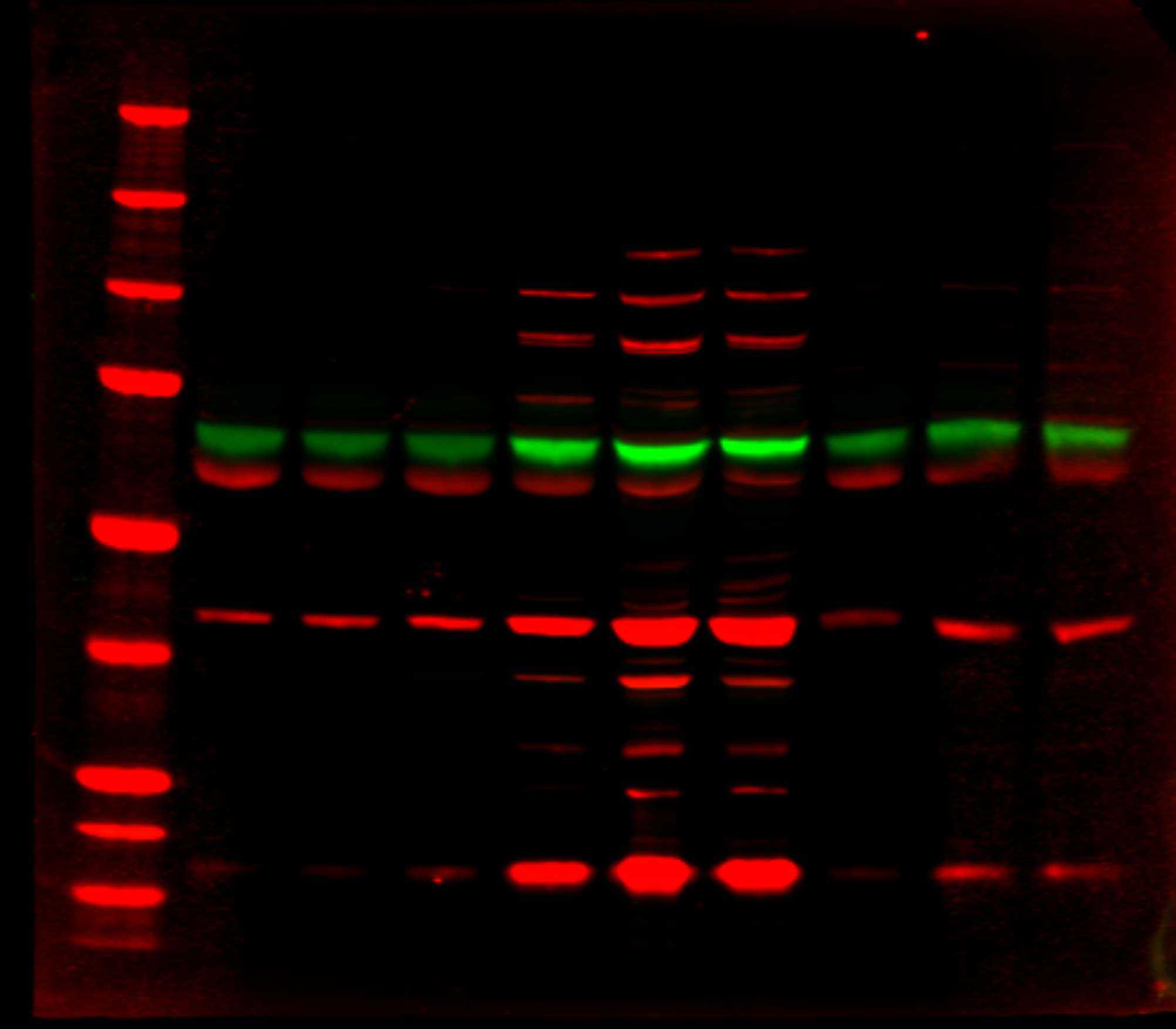

Supplement: Supplementary file 8 — Source Data [file 41467_2022_35252_MOESM8_ESM.zip › Fig1/Fig1a/20190814_0h-72h_Zap70_Dhps_Hyp.tif]

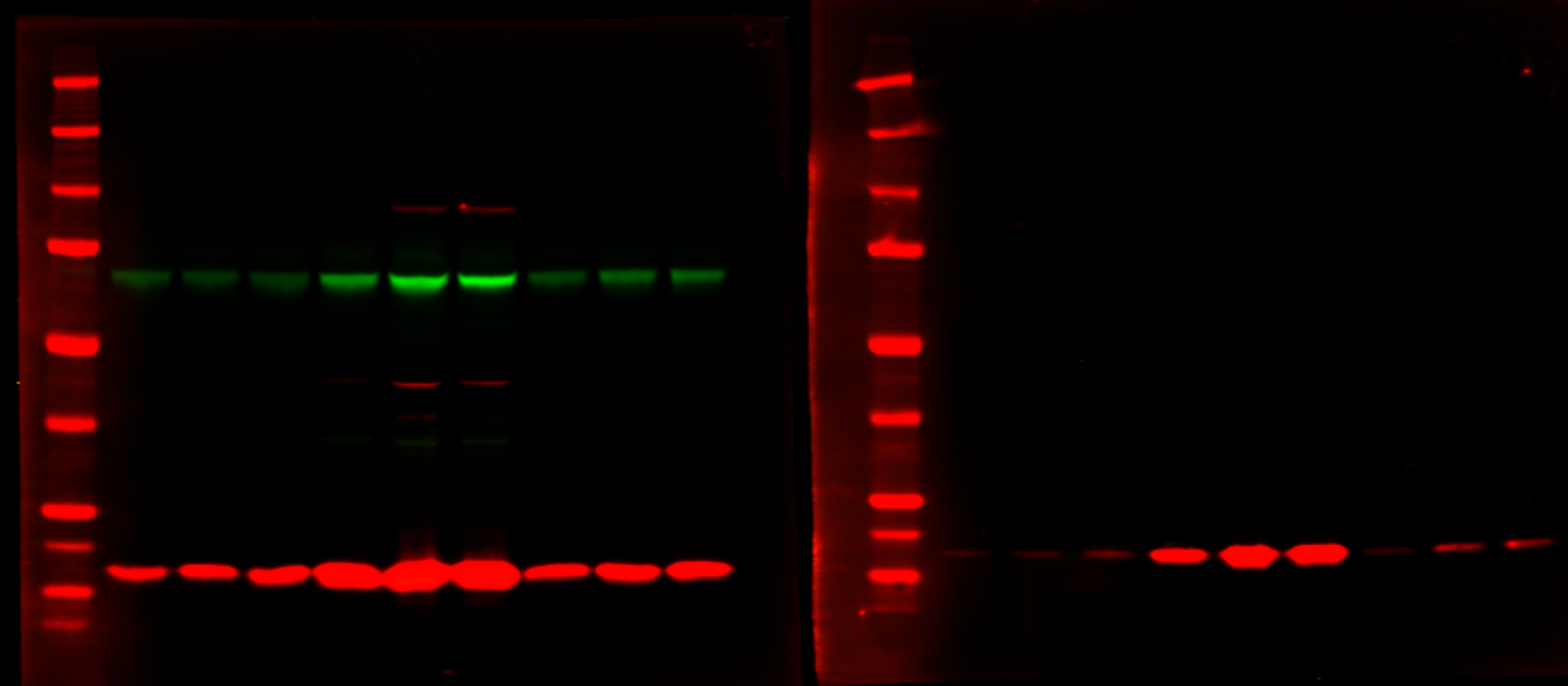

Supplement: Supplementary file 8 — Source Data [file 41467_2022_35252_MOESM8_ESM.zip › Fig1/Fig1a/20190911_0h-72h_Zap70_eIF5a-L_Hyp-mAb-R.tif]

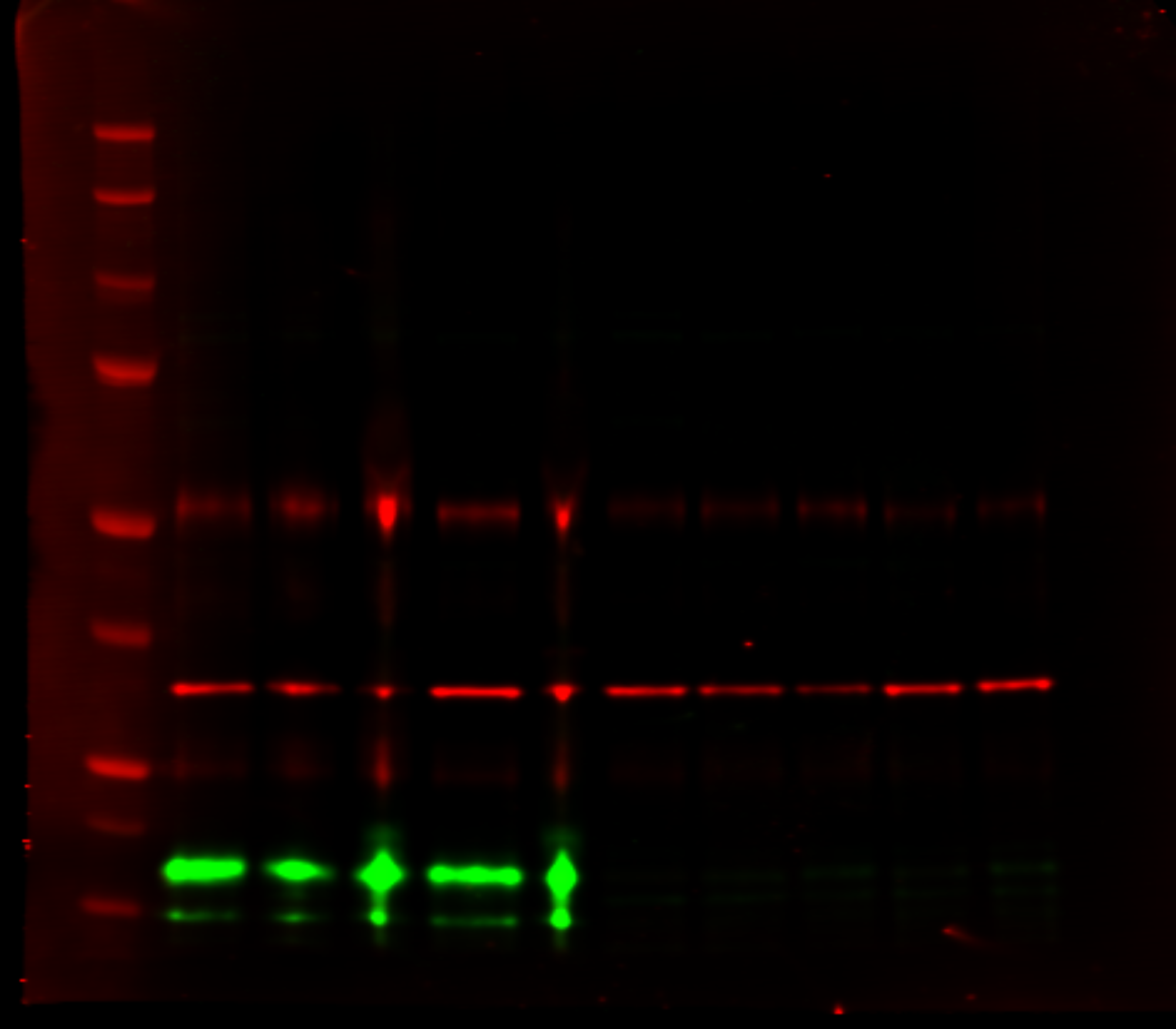

Supplement: Supplementary file 8 — Source Data [file 41467_2022_35252_MOESM8_ESM.zip › Fig2/Fig2b/20200217_sorted-eIF5a_FoxP1-Cdk1-eIF5a.tif]
